# Inhomogeneous and Radiating Composite Fluids

**DOI:** 10.3390/e23111400

**Published:** 2021-10-25

**Authors:** Byron P. Brassel, Sunil D. Maharaj, Rituparno Goswami

**Affiliations:** Astrophysics Research Centre, School of Mathematics, Statistics and Computer Science, University of KwaZulu-Natal, Private Bag X54001, Durban 4000, South Africa; brasselb@ukzn.ac.za (B.P.B.); goswami@ukzn.ac.za (R.G.)

**Keywords:** composite fluids, energy conditions

## Abstract

We consider the energy conditions for a dissipative matter distribution. The conditions can be expressed as a system of equations for the matter variables. The energy conditions are then generalised for a composite matter distribution; a combination of viscous barotropic fluid, null dust and a null string fluid is also found in a spherically symmetric spacetime. This new system of equations comprises the energy conditions that are satisfied by a Type I fluid. The energy conditions for a Type II fluid are also presented, which are reducible to the Type I fluid only for a particular function. This treatment will assist in studying the complexity of composite relativistic fluids in particular self-gravitating systems.

## 1. Introduction

An interesting approach in the study of self-gravitating systems is the idea of complexity. In the past, studies in this approach involved concepts such as entropy and information. A simple and physically quantifiable idea was recently investigated by [1]; it was proposed that relativistic fluids, with homogeneous energy density and isotropic pressure, are characterised with minimal complexity factors. This approach is particularly useful in the study of compact objects and radiating stars. Complexity is encoded in a structure scalar containing components from inhomogeneity, in the energy density, and local anisotropy arising from shear viscosity. Several studies have applied the ideas of Herrera [1] to general relativity [2,3,4,5,6,7,8,9,10,11], and modified gravity theories, especially Einstein–Gauss–Bonnet gravity [12]. Another general concept that may be applied to self-gravitating fluids is energy conditions [13]. Therefore, in this paper we consider the energy conditions for a general composite matter distribution that contains dissipative components in the energy momentum tensor. Our treatment may be applied to different physical scenarios, including dissipative models in relativistic astrophysics. Our results may be helpful in analysing physical quantities associated with complexity in self-gravitating systems.

The energy conditions need to be applied to matter distributions in a gravitational theory for a physically realistic energy momentum tensor. This is an attempt to describe the qualitative features of the matter distribution without having to specify the matter content in an explicit way. Therefore, it is possible to consider physical features in extreme conditions such as gravitational collapse and the occurrence of spacetime singularities without knowledge of the precise form of the matter variables, such as the energy density and the pressure. The energy conditions are described in a comprehensive manner for Type I, II, III and IV fluid distributions by Hawking and Ellis [13] in the context of general relativity. The energy conditions have been used in many studies in cosmology. For example, Santos et al. [14] found bounds on the behaviour of the distance modulus of cosmic sources as a function of redshift for supernovae observations. Santos et al. [15] also studied the energy conditions using type Ia supernovae observations for attractive gravity and cosmic acceleration. The energy conditions have also been utilised in modified gravity theories. Capozziello et al. [16] studied the role of the energy conditions in f(R) gravity. Studies involving the energy conditions have been undertaken in non-minimally coupled f(R,T) gravity [17], symmetric f(Q,T) gravity [18], unimodular F(R,T) gravity [19], and others. In a recent detailed analysis, Kontou and Sanders [20] considered the equations of motion in relation to the energy conditions in general relativity and quantum field theory.

Another important area for application of the energy conditions is relativistic astrophysics. The energy conditions are important in the description of static stars in general relativity; some examples are contained in the treatments [21,22,23,24]. They have also been used in the description of static compact spheres in Einstein–Gauss–Bonnet gravity; some examples are contained in the works [25,26,27]. The energy conditions have been applied in the modeling of radiating stars, which are heat-conducting and undergoing dissipation [28,29,30]. It is important to point out that the energy conditions for a Type I imperfect fluid that conducts heat and radiates energy were first considered by Kolassis et al. [31]. Different forms of the energy conditions for shear-free and shearing spacetimes with heat flux have been used to model dissipative radiating stars in general relativity [32,33,34,35,36,37,38]. We point out that different systems of equations describing the energy conditions are presented in the above treatments, some of which contain errors. It is necessary to provide a general framework in which special cases for particular Type I matter distributions arise simply.

The generalised Santos junction condition was generated by Maharaj et al. [39] by matching the interior geometry of a manifold, containing a barotropic, shear-free heat conducting Type I fluid, to the exterior geometry described by the generalised Vaidya metric, which contains an additional Type II null fluid. An interesting feature of this result is the fact that the pressure of the radiating fluid at the boundary is proportional not only to the heat flux, but also to the non-vanishing energy density of the Type II null string fluid. More recently, the boundary condition for a composite fluid was found by Maharaj and Brassel [40]. It was shown that the pressure at the boundary was proportional to the heat flux, an internal string energy density, anisotropy and the null string energy density of the external Type II fluid. This analysis was extended to include an electromagnetic field in [41]. Solutions to the Einstein field equations with an additional Type II fluid have been studied extensively by [42,43,44]. It is interesting to note that the Type II fluid existing in the exterior region of the radiating star has been studied in isolation without a direct connection to the interior Type I matter field [43,44]. With regards to gravitational collapse, Dawood and Ghosh [45] characterised a large family of solutions to the field equations for a spherically symmetric Type II fluid, and showed that the well known dynamical black hole solutions are a particular subcase of this larger family. These results were then generalised to higher dimensions by Ghosh and Dawood [46]. The generalisation of the Vaidya spacetime arises from the fact that the energy momentum tensor is linear in terms of the mass function. The lesser known Hawking–Ellis Type III and Type IV energy momentum tensors have been studied in detail by [13,47]. It was shown by Maeda and Martínez [47] that these two types of fluids are unphysical due to the fact that they violate the null energy condition and the Type IV fluid admits complex eigenvalues. It is important to note that the authors did not assume time-orientability of the spacetime. Therefore, energy momentum tensors of Type I and II are the most physically relevant. The classification of the energy momentum tensor into four types is possible in arbitrary dimensions N≥4 [48].

The main aim of this study was to find the energy conditions for a generalised composite relativistic fluid, which we show to be of Type I. The resulting generalised conditions may be used in a variety of cosmological models. Note that the new energy conditions generated will be valid in general relativity and *any* other modified theory of gravity. We assume that the spacetime is spherically symmetric. Firstly, we take the matter distribution to be an anisotropic field with a barotropic Type I fluid. The energy conditions are generated as a simple system of seven equations; this system contains all previous treatments of shearing and shear-free heat conducting fluids. Secondly, we assume that the matter distribution is a composite field with a barotropic fluid, null dust and a null string. The energy conditions are found for the composite matter distribution as a more general system of equations. Finally, the energy conditions for a Type II fluid distribution are presented and it is shown that these conditions correspond to a non-diagonalisable energy momentum tensor. In the two appendices, we present the methodology for transforming to the orthonormal basis for Type I and Type II fluids.

## 2. Energy Conditions

Investigating the nature of the energy conditions is an algebraic problem [31] related to the eigenvalue problem of the energy momentum tensor T. In a four-dimensional spacetime manifold, investigating the energy conditions involves solving a quartic polynomial, which is usually difficult and can lead to certain situations where one is faced with complicated analytical expressions of the eigenvalues. This makes the problem difficult to solve in general. In order for a relativistic fluid to be deemed physically reasonable, it should obey the null, weak, dominant and strong energy conditions [13,31,49]. The energy momentum tensor T can be projected onto the orthonormal basis if there exist the orthonormal vectors $E_{0}$, $E_{i}$, with i∈{1,2,3} such that $E_{0}E^{0}<0$ and $E^{i}E_{i}>0$. This is such that the energy momentum tensor is a diagonal (or diagonalisable) matrix of the eigenvalues. If this is the case, then T is a Type I fluid. If the matrix of eigenvalues is *not* diagonalisable, the energy momentum tensor is that of a Type II, III or IV fluid. In this paper, we will analyse all of the energy conditions of both a Type I composite fluid distribution and a Type II fluid, as these correspond to physically relevant matter distributions. Type III and Type IV fluids are unphysical or apply in very special scenarios. For Type I and Type II fluids, the energy conditions are given by:

(i) The null energy condition: For any future pointing null vector k, the total energy density $T_{ab}k^{a}k^{b}\geq 0$. By continuity, for an orthonormal vector E, we then have that $T_{ab}E^{a}E^{b}\geq 0$ at each event on the spacetime.
(ii) The weak energy condition: For any future pointing timelike vector w, the total energy density $T_{ab}w^{a}w^{b}\geq 0$, at each event in the spacetime. The weak energy condition contains the null energy condition.
(iii) The strong energy condition: For any future pointing timelike unit vector w, the stresses of the matter at each event in the spacetime are restricted by the condition $2T_{ab}w^{a}w^{b}+T\geq 0$, where *T* is the trace of the energy momentum tensor **T**.
(iv) The dominant energy condition: For any future pointing timelike or null vector w, the energy density must obey $T_{ab}w^{a}w^{b}\geq 0$ (the *weak energy condition*), and the four-momentum density vector $T_{ab}w^{b}$ must be future pointing and timelike, or null at every event in the spacetime (the *flux energy condition* (The flux energy condition is a weaker form of the dominant energy condition, since no assumption for positive energy densities need be enforced.)) According to any observer, this is to say that the mass-energy flow is always positive and less than the speed of light.

For any astrophysical or cosmological model to be deemed physically reasonable, all four of the above conditions, as well as those of causality, should be obeyed in general.

## 3. Viscous Fluid Distributions

We consider the units G=c=1 and that the spacetime manifold has a Lorentzian signature (−,+,+,+). Viscous fluid distributions arise in several models of radiating stars in which the anisotropy $\pi_{ab}$ is related to the shear $\sigma_{ab}$ by $\pi_{ab}=-2\eta \sigma_{ab}$, for example, see the treatments of [31,32,33,34,35,36,37,38]. We first consider the energy conditions in this type of matter.

### 3.1. Field Equations

In this section we assume that the spacetime geometry is described by the most general spherically symmetric spacetime. In comoving coordinates, the metric is written as:

(1) $$ds^{2}=-A^{2}dt^{2}+B^{2}dr^{2}+C^{2}\left(d\theta^{2}+\sin^{2}\theta d\phi^{2}\right),$$

where the metric functions A=A(r,t), B=B(r,t) and C=C(r,t). The shear tensor $\sigma_{ab}$ is defined as:

(2) $$\sigma_{ab}=u_{\left(a;b\right)}+A_{(a}u_{b)}-\frac{1}{3}\Theta \left(g_{ab}+u_{a}u_{b}\right),$$

where we have:

$$\begin{array}{ccc} A_{a} & = & u_{a;b}u^{b},\\ \Theta & = & u^{a}{}_{;a}, \end{array}$$

as the acceleration vector and expansion scalar, respectively. In the above, the semicolon denotes covariant differentiation and the round brackets on the indices denote symmetrisation. The energy momentum tensor is given by:

(3) $$T_{ab}=\left(\mu +p_{⊥}\right)u_{a}u_{b}+p_{⊥}g_{ab}+\left(p_{\left|\right|}-p_{⊥}\right)X_{a}X_{b}+q_{a}u_{b}+q_{b}u_{a}-2\eta \sigma_{ab},$$

where μ is the energy density, $p_{\left|\right|}$ is the radial pressure, $p_{⊥}$ is the tangential pressure, q is the heat flux vector, X is a four-vector along the radial direction and u is the fluid four-velocity, which is timelike. The quantity η≥0 is the shear viscosity.

Since the coordinates are comoving, we have that

(4) $$u^{a}=\frac{1}{A}\delta^{a}{}_{0},$$

and the two radial vectors

(5) $$q^{a}=q\delta^{a}{}_{1},\;X^{a}=\frac{1}{B}\delta^{a}{}_{1}.$$

These satisfy the following conditions:

$$u^{a}q_{a}=0=X_{a}u^{a},\;X_{a}X^{a}=1,\;u^{a}u_{a}=-1,\;q^{a}q_{a}={\left(qB\right)}^{2}.$$

The expansion scalar is calculated to be

(6) $$\Theta =\frac{1}{A}\left(\frac{\dot{B}}{B}+2\frac{\dot{C}}{C}\right),$$

and the nonvanishing components of the shear tensor (2) are then given by

(7) $$\begin{array}{ccc} \sigma_{11} & = & \frac{2B^{2}}{3A}\left(\frac{\dot{B}}{B}-\frac{\dot{C}}{C}\right), \end{array}$$

(8) $$\begin{array}{ccc} \sigma_{22} & = & -\frac{C^{2}}{3A}\left(\frac{\dot{B}}{B}-\frac{\dot{C}}{C}\right), \end{array}$$

(9) $$\begin{array}{ccc} \sigma_{33} & = & \sin^{2}\theta \sigma_{22}. \end{array}$$

In the above, dots denote derivatives with respect to time. We define the following scalar σ as

(10) $$\left|\sigma \right|=\pm \frac{1}{3A}\left(\frac{\dot{B}}{B}-\frac{\dot{C}}{C}\right),$$

where $\sigma^{2}=\frac{1}{2}\sigma^{ab}\sigma_{ab}$. We can then write

(11) $$\begin{array}{ccc} \sigma^{1}{}_{1} & = & \frac{1}{B^{2}}\sigma_{11}=2\left|\sigma \right|, \end{array}$$

(12) $$\begin{array}{ccc} \sigma^{2}{}_{2} & = & \frac{1}{C^{2}}\sigma_{22}=-\left|\sigma \right|, \end{array}$$

(13) $$\begin{array}{ccc} \sigma^{3}{}_{3} & = & \frac{1}{C^{2}\sin^{2}\theta }\sigma_{33}=-\left|\sigma \right|, \end{array}$$

so that $\sigma^{a}{}_{a}=0$ and the shear tensor is trace-free.

The nonzero components of the energy momentum tensor (3) are then

(14) $$\begin{array}{ccc} T_{00} & = & \mu A^{2}, \end{array}$$

(15) $$\begin{array}{ccc} T_{01} & = & -qAB^{2}, \end{array}$$

(16) $$\begin{array}{ccc} T_{11} & = & B^{2}\left(p_{\left|\right|}-2\eta \sigma^{1}{}_{1}\right), \end{array}$$

(17) $$\begin{array}{ccc} T_{22} & = & C^{2}\left(p_{⊥}-2\eta \sigma^{2}{}_{2}\right), \end{array}$$

(18) $$\begin{array}{ccc} T_{33} & = & \sin^{2}\theta T_{22}, \end{array}$$

which follow from (3). The nonzero Einstein tensor components are given by

(19) $$\begin{array}{ccc} G_{00} & = & 2\frac{\dot{B}\dot{C}}{BC}+\frac{A^{2}}{C^{2}}+\frac{{\dot{C}}^{2}}{C^{2}}-\frac{A^{2}}{B^{2}}\left(2\frac{C^{\prime \prime }}{C}+\frac{C^{\prime 2}}{C^{2}}-2\frac{B^{\prime }C^{\prime }}{BC}\right), \end{array}$$

(20) $$\begin{array}{ccc} G_{01} & = & 2\left(-\frac{{\dot{C}}^{\prime }}{C}+\frac{\dot{B}C^{\prime }}{BC}+\frac{A^{\prime }\dot{C}}{AC}\right), \end{array}$$

(21) $$\begin{array}{ccc} G_{11} & = & \frac{B^{2}}{A^{2}}\left(-2\frac{\ddot{C}}{C}-\frac{{\dot{C}}^{2}}{C^{2}}+2\frac{\dot{A}\dot{C}}{AC}\right)+\frac{C^{\prime 2}}{C^{2}}+2\frac{A^{\prime }C^{\prime }}{AC}-\frac{B^{2}}{C^{2}}, \end{array}$$

(22) $$\begin{array}{ccc} G_{22} & = & -\frac{C^{2}}{A^{2}}\left(\frac{\ddot{B}}{B}-\frac{\dot{A}\dot{B}}{AB}+\frac{\dot{B}\dot{C}}{BC}-\frac{\dot{A}\dot{C}}{AC}+\frac{\ddot{C}}{C}\right)+\frac{C^{2}}{B^{2}}\left(\frac{A^{\prime \prime }}{A}-\frac{A^{\prime }B^{\prime }}{AB}+\frac{A^{\prime }C^{\prime }}{AC}-\frac{B^{\prime }C^{\prime }}{BC}+\frac{C^{\prime \prime }}{C}\right), \end{array}$$

(23) $$\begin{array}{ccc} G_{33} & = & \sin^{2}\theta G_{22}, \end{array}$$

for the metric (1). In the above, primes denote differentiation with respect to the radial coordinate *r*. The Einstein field equations $G_{ab}=8\pi T_{ab}$ can then be written, with the aid of (11)–(13), as:

(24) $$\begin{array}{ccc} 8\pi \mu & = & \frac{2}{A^{2}}\frac{\dot{B}\dot{C}}{BC}+\frac{1}{C^{2}}+\frac{1}{A^{2}}\frac{{\dot{C}}^{2}}{C^{2}}-\frac{1}{B^{2}}\left(2\frac{C^{\prime \prime }}{C}+\frac{C^{\prime 2}}{C^{2}}-2\frac{B^{\prime }C^{\prime }}{BC}\right), \end{array}$$

(25) $$\begin{array}{ccc} 8\pi \left(p_{\left|\right|}-4\eta \left|\sigma \right|\right) & = & \frac{1}{A^{2}}\left(-2\frac{\ddot{C}}{C}-\frac{{\dot{C}}^{2}}{C^{2}}+2\frac{\dot{A}\dot{C}}{AC}\right)+\frac{1}{B^{2}}\left(\frac{C^{\prime 2}}{C^{2}}+2\frac{A^{\prime }C^{\prime }}{AC}\right)-\frac{1}{C^{2}}, \end{array}$$

(26) $$\begin{array}{ccc} 8\pi \left(p_{⊥}+2\eta \left|\sigma \right|\right) & = & -\frac{1}{A^{2}}\left(\frac{\ddot{B}}{B}-\frac{\dot{A}\dot{B}}{AB}+\frac{\dot{B}\dot{C}}{BC}-\frac{\dot{A}\dot{C}}{AC}+\frac{\ddot{C}}{C}\right)+\frac{1}{B^{2}}\left(\frac{A^{\prime \prime }}{A}-\frac{A^{\prime }B^{\prime }}{AB}+\frac{A^{\prime }C^{\prime }}{AC}-\frac{B^{\prime }C^{\prime }}{BC}+\frac{C^{\prime \prime }}{C}\right), \end{array}$$

(27) $$\begin{array}{ccc} 8\pi q & = & -\frac{2}{AB^{2}}\left(-\frac{{\dot{C}}^{\prime }}{C}+\frac{\dot{B}C^{\prime }}{BC}+\frac{A^{\prime }\dot{C}}{AC}\right), \end{array}$$

for the spherically symmetric metric (1) and the anisotropic, heat-conducting matter distribution (3).

### 3.2. Eigenvalues

In order to write down the energy conditions, the eigenvalues of the energy momentum tensor need to be calculated. For Type I fluids, the eigenvalues must be strictly real [31,47,50]. If $\lambda_{0}$ denotes the eigenvalue corresponding to the timelike eigenvector, then for the Type I energy momentum tensor, we have the following relations:

1. Null energy conditions:
  (28) $$-\lambda_{0}+\lambda_{i}\geq 0,\;i\in \left\{1,2,3\right\}.$$
2. Weak energy conditions:
  (29) $$-\lambda_{0}\geq 0,\;-\lambda_{0}+\lambda_{i}\geq 0,\;i\in \left\{1,2,3\right\}.$$
3. Dominant energy conditions:
  (30) $$-\lambda_{0}\geq 0,\;\lambda_{0}\leq \lambda_{i}\leq -\lambda_{0},\;i\in \left\{1,2,3\right\}.$$
4. Strong energy conditions:
  (31) $$-\lambda_{0}+\sum_{i=1}^{3}\lambda_{i}\geq 0,\;-\lambda_{0}+\lambda_{i}\geq 0,\;i\in \left\{1,2,3\right\}.$$

It can be seen from (30) and (31) that the dominant and strong energy conditions, respectively, imply the weak energy condition. The weak energy condition (29) also implies the null energy condition (28). The eigenvalues λ of the energy momentum tensor are the roots of the following equation:

(32) $$|T_{ab}-\lambda g_{ab}|=0,$$

which holds in a general spacetime.

Different forms of the energy conditions in spherical symmetry are given in several treatments, some of which contain errors (see for example Pinheiro and Chan [37] and Pinheiro and Chan [38]). We therefore present the correct expressions for the energy conditions, for an imperfect fluid with shearing stresses. In spherical symmetry using Expressions (1)–(3) we can write (32) in the form

(33) $$\begin{array}{c} \left|\begin{array}{cccc} A^{2}\left(\mu +\lambda \right) & -\bar{q}AB & 0 & 0\\ -\bar{q}AB & B^{2}\left(p_{\left|\right|}-\lambda -2\eta \sigma^{1}{}_{1}\right) & 0 & 0\\ 0 & 0 & C^{2}\left(p_{⊥}-\lambda -2\eta \sigma^{2}{}_{2}\right) & 0\\ 0 & 0 & 0 & C^{2}\sin^{2}\theta \left(p_{⊥}-\lambda -2\eta \sigma^{2}{}_{2}\right) \end{array}\right|=0, \end{array}$$

with $\bar{q}=qB$. We note the presence of the $\sin^{2}\theta$ term, which was incorrectly omitted from the work of Pinheiro and Chan [37]. This omission has several consequences with regards to the final result. Calculating the determinant of the above equation gives

(34) $$\left[\lambda^{2}+\left(\mu -p_{\left|\right|}\right)\lambda +{\bar{q}}^{2}-\mu p_{\left|\right|}+2\left(\mu +\lambda \right)\eta \sigma^{1}{}_{1}\right]{\left(p_{⊥}-\lambda -2\eta \sigma^{2}{}_{2}\right)}^{2}\left(-A^{2}B^{2}C^{4}\sin^{2}\theta \right)=0,$$

which is different from [37]. One of the solutions of (34) can be written as:

(35) $$\lambda^{2}+\left(\mu -p_{\left|\right|}\right)\lambda +{\bar{q}}^{2}-\mu p_{\left|\right|}+2\left(\mu +\lambda \right)\eta \sigma^{1}{}_{1}=0.$$

The two roots of (35) are given by:

(36) $$\begin{array}{ccc} \lambda_{0} & = & -\frac{1}{2}\left[\mu -p_{\left|\right|}+2\eta \sigma^{1}{}_{1}+\Delta \right], \end{array}$$

(37) $$\begin{array}{ccc} \lambda_{1} & = & -\frac{1}{2}\left[\mu -p_{\left|\right|}+2\eta \sigma^{1}{}_{1}-\Delta \right], \end{array}$$

where:

(38) $$\Delta^{2}={\left(\mu +p_{\left|\right|}-2\eta \sigma^{1}{}_{1}\right)}^{2}-4{\bar{q}}^{2}.$$

In the above, Δ>0 in order to have real roots. Also note that if Δ=0, the energy momentum tensor (3) is a Type II fluid. Otherwise it is Type I. This equation can then be written as

(39) $$\left|\mu +p_{\left|\right|}-2\eta \sigma^{1}{}_{1}\right|-2|\bar{q}|\geq 0.$$

The second solution of (34) is

(40) $${\left(p_{⊥}-\lambda -2\eta \sigma^{2}{}_{2}\right)}^{2}=0,$$

which has the two roots

(41) $$\lambda_{2,3}=p_{⊥}-2\eta \sigma^{2}{}_{2},$$

which are repeated. We note that the four roots (36), (37) and (41) are corrections to those given in [37].

### 3.3. Energy Conditions

We are now in the position to present the four different forms of the energy conditions. The condition Δ>0 applies in all cases.

#### 3.3.1. Null Energy Conditions (NEC)

Using Equations (28) and the roots (36), (37) and (41), the null energy conditions become

(42) $$\begin{array}{ccc} \mu +p_{\left|\right|}-2\eta \sigma^{1}{}_{1}+\Delta & \geq & 0, \end{array}$$

(43) $$\begin{array}{ccc} \mu -p_{\left|\right|}+2\eta \sigma^{1}{}_{1}+2\left(p_{⊥}-2\eta \sigma^{2}{}_{2}\right)+\Delta & \geq & 0, \end{array}$$

(44) $$\begin{array}{ccc} \Delta & > & 0. \end{array}$$

#### 3.3.2. Weak Energy Conditions (WEC)

Using Equations (29) and the roots (36), (37) and (41) we find that the weak energy conditions become

(45) $$\begin{array}{ccc} \mu -p_{\left|\right|}+2\eta \sigma^{1}{}_{1}+\Delta & \geq & 0, \end{array}$$

(46) $$\begin{array}{ccc} \mu -p_{\left|\right|}+2\eta \sigma^{1}{}_{1}+2\left(p_{⊥}-2\eta \sigma^{2}{}_{2}\right)+\Delta & \geq & 0, \end{array}$$

(47) $$\begin{array}{ccc} \Delta & > & 0. \end{array}$$

It can clearly be seen that the weak energy conditions contain the null energy conditions (42)–(44).

#### 3.3.3. Dominant Energy Conditions (DEC)

Using (30) along with the roots (36), (37) and (41), the dominant energy conditions are then

(48) $$\begin{array}{ccc} \mu -p_{\left|\right|}+2\eta \sigma^{1}{}_{1} & \geq & 0, \end{array}$$

(49) $$\begin{array}{ccc} \mu -p_{\left|\right|}+2\eta \sigma^{1}{}_{1}\pm 2\left(p_{⊥}-2\eta \sigma^{2}{}_{2}\right)+\Delta & \geq & 0, \end{array}$$

(50) $$\begin{array}{ccc} \Delta & > & 0. \end{array}$$

It can clearly be seen that (50) is analogous with (47). The dominant energy conditions imply the weak energy conditions.

#### 3.3.4. Strong Energy Conditions (SEC)

Using (31) and completing the sum with i∈{1,2,3} as well as the four roots (36), (37) and (41), the strong energy conditions then become

(51) $$\begin{array}{ccc} 2\left(p_{⊥}-2\eta \sigma^{2}{}_{2}\right)+\Delta & \geq & 0, \end{array}$$

(52) $$\begin{array}{ccc} \mu -p_{\left|\right|}+2\eta \sigma^{1}{}_{1}+2\left(p_{⊥}-2\eta \sigma^{2}{}_{2}\right)+\Delta & \geq & 0, \end{array}$$

(53) $$\begin{array}{ccc} \Delta & > & 0, \end{array}$$

where (52) and (53) are the same as the weak energy conditions (46) and (47).

We note that the energy conditions (45)–(47), (48)–(50) and (51)–(53) contain corrections to those found by [37].

#### 3.3.5. Summary of the Energy Conditions

A summary of the energy conditions is given below:

(54) $$\begin{array}{ccc} \mu +p_{\left|\right|}-4\eta \left|\sigma \right|+\Delta & \geq & 0, \end{array}$$

(55) $$\begin{array}{ccc} \mu -p_{\left|\right|}+4\eta \left|\sigma \right|+\Delta & \geq & 0, \end{array}$$

(56) $$\begin{array}{ccc} \mu -p_{\left|\right|}+8\eta \left|\sigma \right|+2p_{⊥}+\Delta & \geq & 0, \end{array}$$

(57) $$\begin{array}{ccc} \mu -p_{\left|\right|}+4\eta \left|\sigma \right| & \geq & 0, \end{array}$$

(58) $$\begin{array}{ccc} \mu -p_{\left|\right|}-2p_{⊥}+\Delta & \geq & 0, \end{array}$$

(59) $$\begin{array}{ccc} 2\left(p_{⊥}+4\eta \left|\sigma \right|\right)+\Delta & \geq & 0, \end{array}$$

(60) $$\begin{array}{ccc} \Delta & > & 0, \end{array}$$

where we have utilised (11)–(13) and where

(61) $$\Delta =\sqrt{{\left(\mu +p_{\left|\right|}-4\eta \left|\sigma \right|\right)}^{2}-4{\bar{q}}^{2}}.$$

These apply for a spherically symmetric fluid with a Type I energy momentum (3). We expect that the conditions should be satisfied, for example, in a radiating collapsing star with dissipative heat fluxes.

We can now state the following theorem:

**Theorem** **1.** *Consider a four-dimensional spacetime M described by the general spherically symmetric metric (1) with an anisotropic Type I matter distribution containing a barotropic fluid. In order for the null, weak, dominant and energy conditions to be satisfied, such a fluid distribution must fulfil the conditions given in (54)–(60) with Δ>0.*

We can now regain the energy conditions for a perfect fluid matter distribution from (54)–(60). If we set η=0 and $p_{\left|\right|}=p_{⊥}=p$ then we find that (54)–(60) reduces to the following special cases:

- Null energy conditions:
  μ+p≥0,μ−p≥0,
- Weak energy conditions:
  μ≥0,μ+p≥0,
- Dominant energy conditions:
  μ≥|p|,
- Strong energy conditions:
  μ+p≥0,μ+3p≥0,

which are the energy conditions for a perfect fluid in general relativity.

The energy conditions are important for the description of dissipation effects in a radiating relativistic star, including transport processes at the stellar surface. However, it is important to note that the energy conditions may be violated under certain circumstances in astrophysics and cosmology. This is true for theories in modified gravity [51], extended theories of gravity [52], quantum field theories [20] and branes [53]. For a general discussion on the violation of energy conditions see Barcelo and Visser [54].

In addition, observe that we have used the relationship for the anisotropic stress tensor $\pi_{ab}=-2\eta \sigma_{ab}$ in the definition (3). This is the standard approach but it is limited corresponding to standard irreversible thermodynamics. It is possible that this definition involving the shear viscosity may lead to the violation of causality in extreme conditions. To avoid this situation we need to employ a causal dissipative theory. Herrera et al. [55] have considered the general framework for a general spherically symmetric spacetime with causal thermodynamics.

## 4. Composite Fluid Distributions

In this section, we consider the spacetime to be the general shearing metric (1). It is then possible to generate energy conditions for Type I matter fields that are more general than in Section 3.3. We now describe a matter distribution that is a composite of barotropic matter, null dust and a null string fluid. Distributions of this kind have been considered by Kiselev [56], Heydarzade and Darabi [57] and Brassel and Maharaj [58] in general relativity and Einstein–Gauss–Bonnet gravity to describe cosmological radiating fields sourced by a Vaidya-like radiating metric. Composite matter fields may also be used to describe the interior of radiating collapsing stars in general relativity, as shown by Di Prisco et al. [59] and Maharaj and Brassel [40,41].

### 4.1. Field Equations

The energy momentum tensor is taken to be a generalised imperfect composite fluid of the form

(62) $$\begin{array}{ccc} T_{ab} & = & \left(\mu +p_{⊥}\right)u_{a}u_{b}+p_{⊥}g_{ab}+\left(p_{\left|\right|}-p_{⊥}\right)X_{a}X_{b}+q_{a}u_{b}+q_{b}u_{a}-2\eta \sigma_{ab}\\ & & +\epsilon l_{a}l_{b}+\left(\rho +P\right)\left(l_{a}n_{b}+l_{b}n_{a}\right)+Pg_{ab}, \end{array}$$

where μ is the energy density, $p_{\left|\right|}$ is the radial pressure, $p_{⊥}$ is the tangential pressure, q is the heat flux vector, X is a four-vector along the radial direction and u is the fluid four-velocity, η≥0 is the shear viscosity and $\sigma_{ab}$ is the shear tensor. We also have that ϵ is the energy density of the null dust, ρ is the null string energy density and P is the pressure of the null string fluid. The vectors l and n are null. We then have the following relations:

$$\begin{array}{ccc} u^{a}q_{a} & = & 0,\;X_{a}X^{a}=1,\;u^{a}u_{a}=-1,\\ l^{a}l_{a}=n^{a}n_{a} & = & 0,\;l_{a}n^{a}=-1,\;l^{a}u_{a}=-1, \end{array}$$

which lead to the particular forms:

$$\begin{array}{ccc} u^{a} & = & \frac{1}{A}\delta^{a}{}_{0},\;q^{a}=q\delta^{a}{}_{1},\;X^{a}=\frac{1}{B}\delta^{a}{}_{1},\\ l^{a} & = & \frac{1}{A}\delta^{a}{}_{0}+\frac{1}{B}\delta^{a}{}_{1},\;n^{a}=\frac{1}{2A}\delta^{a}{}_{0}+\frac{1}{2B}\delta^{a}{}_{1}. \end{array}$$

The nonzero components of the energy momentum tensor (62) are then given by:

(63) $$\begin{array}{ccc} T_{00} & = & A^{2}\left(\mu +\epsilon +\rho \right), \end{array}$$

(64) $$\begin{array}{ccc} T_{01} & = & -AB^{2}\left(q+\frac{1}{B}\epsilon \right), \end{array}$$

(65) $$\begin{array}{ccc} T_{11} & = & B^{2}\left(p_{\left|\right|}+\epsilon -\rho -2\eta \sigma^{1}{}_{1}\right), \end{array}$$

(66) $$\begin{array}{ccc} T_{22} & = & C^{2}\left(p_{⊥}+P-2\eta \sigma^{2}{}_{2}\right), \end{array}$$

(67) $$\begin{array}{ccc} T_{33} & = & \sin^{2}\theta T_{22}, \end{array}$$

which reduce to (14)–(18) for barotropic matter. The Einstein field equations $G_{ab}=8\pi T_{ab}$ then become:

(68) $$\begin{array}{ccc} 8\pi \left(\mu +\epsilon +\rho \right) & = & \frac{2}{A^{2}}\frac{\dot{B}\dot{C}}{BC}+\frac{1}{C^{2}}+\frac{1}{A^{2}}\frac{{\dot{C}}^{2}}{C^{2}}-\frac{1}{B^{2}}\left(2\frac{C^{\prime \prime }}{C}+\frac{C^{\prime 2}}{C^{2}}-2\frac{B^{\prime }C^{\prime }}{BC}\right), \end{array}$$

(69) $$\begin{array}{ccc} 8\pi \left(p_{\left|\right|}+\epsilon -\rho -4\eta \left|\sigma \right|\right) & = & \frac{1}{A^{2}}\left(-2\frac{\ddot{C}}{C}-\frac{{\dot{C}}^{2}}{C^{2}}+2\frac{\dot{A}\dot{C}}{AC}\right)+\frac{1}{B^{2}}\left(\frac{C^{\prime 2}}{C^{2}}+2\frac{A^{\prime }C^{\prime }}{AC}\right)-\frac{1}{C^{2}},\\ 8\pi \left(p_{⊥}+P+2\eta \left|\sigma \right|\right) & = & -\frac{1}{A^{2}}\left(\frac{\ddot{B}}{B}-\frac{\dot{A}\dot{B}}{AB}+\frac{\dot{B}\dot{C}}{BC}-\frac{\dot{A}\dot{C}}{AC}+\frac{\ddot{C}}{C}\right) \end{array}$$

(70) $$\begin{array}{ccc} & & +\frac{1}{B^{2}}\left(\frac{A^{\prime \prime }}{A}-\frac{A^{\prime }B^{\prime }}{AB}+\frac{A^{\prime }C^{\prime }}{AC}-\frac{B^{\prime }C^{\prime }}{BC}+\frac{C^{\prime \prime }}{C}\right), \end{array}$$

(71) $$\begin{array}{ccc} 8\pi \left(q+\frac{1}{B}\epsilon \right) & = & -\frac{2}{AB^{2}}\left(-\frac{{\dot{C}}^{\prime }}{C}+\frac{\dot{B}C^{\prime }}{BC}+\frac{A^{\prime }\dot{C}}{AC}\right), \end{array}$$

where we have utilised (11)–(13).

### 4.2. Eigenvalues

The determinant equation

$$|T_{ab}-\lambda g_{ab}|=0$$

then becomes, using (1), (2) and (62),

(72) $$\begin{array}{c} \left|\begin{array}{cccc} A^{2}\left(\mu +\epsilon +\rho +\lambda \right) & -\tilde{q}AB & 0 & 0\\ -\tilde{q}AB & B^{2}\left(p_{\left|\right|}+\epsilon -\rho -\lambda -2\eta \sigma^{1}{}_{1}\right) & 0 & 0\\ 0 & 0 & C^{2}\left(p_{⊥}+P-\lambda -2\eta \sigma^{2}{}_{2}\right) & 0\\ 0 & 0 & 0 & C^{2}\sin^{2}\theta \left(p_{⊥}+P-\lambda -2\eta \sigma^{2}{}_{2}\right) \end{array}\right|=0, \end{array}$$

with $\tilde{q}=qB+\epsilon$. The above determinant equation reduces to the following quartic equation in λ,

(73) $$\begin{array}{c} \left[\lambda^{2}+\left(\mu -p_{\left|\right|}+2\rho \right)\lambda +{\tilde{q}}^{2}-\left(\mu +\epsilon +\rho \right)\left(p_{\left|\right|}+\epsilon -\rho \right)+2\left(\mu +\epsilon +\rho +\lambda \right)\eta \sigma^{1}{}_{1}\right]\\ \times {\left(p_{⊥}+P-\lambda -2\eta \sigma^{2}{}_{2}\right)}^{2}\left(-A^{2}B^{2}C^{4}\sin^{2}\theta \right)=0. \end{array}$$

Note that if ϵ=ρ=P=0 then (73) becomes (34) for barotropic matter. Since $-A^{2}B^{2}C^{4}\sin^{2}\theta \neq 0$, one solution to (73) is given by

(74) $$\lambda^{2}+\left(\mu -p_{\left|\right|}+2\rho \right)\lambda +{\tilde{q}}^{2}-\left(\mu +\epsilon +\rho \right)\left(p_{\left|\right|}+\epsilon -\rho \right)+2\left(\mu +\epsilon +\rho +\lambda \right)\eta \sigma^{1}{}_{1}=0.$$

This is a second order polynomial in λ and yields the two roots

(75) $$\begin{array}{ccc} \lambda_{0} & = & -\frac{1}{2}\left[\mu -p_{\left|\right|}+2\rho +2\eta \sigma^{1}{}_{1}+\Delta \right], \end{array}$$

(76) $$\begin{array}{ccc} \lambda_{1} & = & -\frac{1}{2}\left[\mu -p_{\left|\right|}+2\rho +2\eta \sigma^{1}{}_{1}-\Delta \right], \end{array}$$

where:

(77) $$\Delta^{2}=\left(\mu +p_{\left|\right|}+2\epsilon -2\eta \sigma^{1}{}_{1}\right)-4{\tilde{q}}^{2}.$$

We require Δ>0 in order for roots to be real and for the fluid to be of Type I. This implies

(78) $$\left|\mu +p_{\left|\right|}+2\epsilon -2\eta \sigma^{1}{}_{1}\right|-2|\tilde{q}|\geq 0.$$

We note the presence of the null dust term ϵ in (78), which does not appear in earlier treatments. In the above inequality (78), we observe that for very large $\tilde{q}=qB+\epsilon$, the second term in the modulus sign will dominate and the matter field will become a Type IV fluid. Alternatively, if the shear viscosity η is large enough, the first term within the modulus sign may also become negative, and the composite matter field will become Type IV, and the energy momentum tensor (62) will become unphysical, specifically for a stellar configuration of matter. The second solution of (73) is given by

$${\left(p_{⊥}+P-\lambda -2\eta \sigma^{2}{}_{2}\right)}^{2}=0,$$

which yields the repeated roots

(79) $$\begin{array}{ccc} \lambda_{2,3} & = & p_{⊥}+P-2\eta \sigma^{2}{}_{2}. \end{array}$$

It is important to note the presence of the null dust and null string fluid components (ϵ and ρ respectively), and the null string pressure P in the above expressions. These equations are generalisations of those found by Kolassis et al. [31] for a Type I fluid.

### 4.3. Energy Conditions

We can now present the energy conditions for a composite fluid distribution.

#### 4.3.1. Null Energy Conditions (NEC)

Utilising (28) along with the solutions (75), (76) and (79), the null energy conditions take the form:

(80) $$\begin{array}{ccc} \mu +p_{\left|\right|}+2\epsilon -2\eta \sigma^{1}{}_{1}+\Delta & \geq & 0, \end{array}$$

(81) $$\begin{array}{ccc} \mu -p_{\left|\right|}+2\rho +2\eta \sigma^{1}{}_{1}+2\left(p_{⊥}+P-2\eta \sigma^{2}{}_{2}\right)+\Delta & \geq & 0, \end{array}$$

(82) $$\begin{array}{ccc} \Delta & > & 0. \end{array}$$

#### 4.3.2. Weak Energy Conditions (WEC)

Using (29) along with (75), (76) and (79), we can write the weak energy conditions as:

(83) $$\begin{array}{ccc} \mu -p_{\left|\right|}+2\rho +2\eta \sigma^{1}{}_{1}+\Delta & \geq & 0, \end{array}$$

(84) $$\begin{array}{ccc} \mu -p_{\left|\right|}+2\rho +2\eta \sigma^{1}{}_{1}+2\left(p_{⊥}+P-2\eta \sigma^{2}{}_{2}\right)+\Delta & \geq & 0, \end{array}$$

(85) $$\begin{array}{ccc} \Delta & > & 0. \end{array}$$

#### 4.3.3. Dominant Energy Conditions (DEC)

Making use of (30) and the solutions (75), (76) and (79), the dominant energy conditions can be expressed as:

(86) $$\begin{array}{ccc} \mu -p_{\left|\right|}+2\rho +2\eta \sigma^{1}{}_{1} & \geq & 0, \end{array}$$

(87) $$\begin{array}{ccc} \mu -p_{\left|\right|}+2\rho +2\eta \sigma^{1}{}_{1}\pm 2\left(p_{⊥}+P-2\eta \sigma^{2}{}_{2}\right)+\Delta & \geq & 0, \end{array}$$

(88) $$\begin{array}{ccc} \Delta & > & 0. \end{array}$$

The dominant energy conditions imply the weak energy conditions for the stress-energy tensor (62).

#### 4.3.4. Strong Energy Conditions (SEC)

Using (75), (76) and (79), and evaluating the sum with i∈{1,2,3} in (31), the strong energy conditions are:

(89) $$\begin{array}{ccc} 2\left(p_{⊥}+P-2\eta \sigma^{2}{}_{2}\right)+\Delta & \geq & 0, \end{array}$$

(90) $$\begin{array}{ccc} \mu -p_{\left|\right|}+2\rho +2\eta \sigma^{1}{}_{1}+2\left(p_{⊥}+P-2\eta \sigma^{2}{}_{2}\right)+\Delta & \geq & 0, \end{array}$$

(91) $$\begin{array}{ccc} \Delta & > & 0. \end{array}$$

In the above, (90) and (91) are identical to the weak energy conditions (84) and (85).

#### 4.3.5. Summary of the Energy Conditions

Summarising our results, with the aid of (11)–(13), the energy conditions can be written as:

(92) $$\begin{array}{ccc} \mu +p_{\left|\right|}+2\epsilon -4\eta \left|\sigma \right|+\Delta & \geq & 0, \end{array}$$

(93) $$\begin{array}{ccc} \mu -p_{\left|\right|}+2\rho +4\eta \left|\sigma \right|+\Delta & \geq & 0, \end{array}$$

(94) $$\begin{array}{ccc} \mu -p_{\left|\right|}+2\rho +8\eta \left|\sigma \right|+2\left(p_{⊥}+P\right)+\Delta & \geq & 0, \end{array}$$

(95) $$\begin{array}{ccc} \mu -p_{\left|\right|}+2\rho +4\eta \left|\sigma \right| & \geq & 0, \end{array}$$

(96) $$\begin{array}{ccc} \mu -p_{\left|\right|}+2\rho -2\left(p_{⊥}+P\right)+\Delta & \geq & 0, \end{array}$$

(97) $$\begin{array}{ccc} 2\left(p_{⊥}+P+4\eta \left|\sigma \right|\right)+\Delta & \geq & 0, \end{array}$$

(98) $$\begin{array}{ccc} \Delta & > & 0, \end{array}$$

and where

(99) $$\Delta =\sqrt{{\left(\mu +p_{\left|\right|}+2\epsilon -4\eta \left|\sigma \right|\right)}^{2}-4{\tilde{q}}^{2}},$$

with $\tilde{q}=qB+\epsilon$. The system (92)–(98) comprises the generalised energy conditions for the general spherically symmetric metric (1) for a composite matter distribution. They reduce to the system (54)–(60) in the absence of null dust and the null string fluid components (ϵ and ρ, respectively), and the null string pressure P, which are present in (62).

We can now state the following theorem:

**Theorem** **2.** *Consider a four-dimensional spacetime M described by the general spherically symmetric metric (1) with an anisotropic Type I matter field (where Δ>0) containing a combination of a barotropic fluid, null dust and a null string fluid. In order for the null, weak, dominant and strong energy conditions to be satisfied, such a fluid distribution must fulfill the conditions given in (92)–(98).*

As mentioned earlier, if Δ=0 the energy momentum tensor becomes a Type II fluid. The above energy conditions (92)–(98) then reduce to the following:

1. NEC:
  (100) $$\begin{array}{ccc} \mu +p_{\left|\right|}+2\epsilon -4\eta \left|\sigma \right| & \geq & 0, \end{array}$$
  (101) $$\begin{array}{ccc} \mu -p_{\left|\right|}+2\rho +8\eta \left|\sigma \right|+2\left(p_{⊥}+P\right) & \geq & 0. \end{array}$$
2. WEC:
  (102) $$\mu -p_{\left|\right|}+2\rho +4\eta \left|\sigma \right|\geq 0,$$
  along with system (100) and (101).
3. DEC:
  (103) $$\mu -p_{\left|\right|}+2\rho -2\left(p_{⊥}+P\right)\geq 0,$$
  along with system (100) and (101).
4. SEC:
  (104) $$p_{⊥}+P+2\eta \left|\sigma \right|\geq 0,$$
  in addition to system (100) and (101).

When ϵ=ρ=0 these will reduce accordingly to the barotropic fluid case.

## 5. Energy Conditions of a Two-Component Fluid of Null Dust and a Null String

Solutions of Einstein’s field equations with an additional Type II fluid were studied in detail by Wang and Wu [42]. These were further extended by Brassel et al. [43] for various equations of state. The generalised Vaidya metric in single (exploding) null coordinates (v,r,θ,ϕ) is given as

$$ds^{2}=-\left(1-\frac{2m(v,r)}{r}\right)dv^{2}+2\varepsilon dvdr+r^{2}\left(d\theta^{2}+\sin^{2}\theta d\phi^{2}\right),$$

where ε=±1. The function m(v,r) describes the Misner–Sharp mass and can be obtained by integration of the Einstein field equations with combinations of perfect string fluid and null matter sources. When ε=−1 the null coordinate *v* represents retarded Eddington time and there is outgoing radiation. When ε=+1 the coordinate *v* represents the Eddington advanced time and we have ingoing/collapsing radiation.

The Einstein tensor components are:

(105) $$\begin{array}{ccc} G^{0}{}_{0} & = & G^{1}{}_{1}=-\frac{2m_{r}}{r^{2}}, \end{array}$$

(106) $$\begin{array}{ccc} G^{1}{}_{0} & = & \frac{2m_{v}}{r^{2}}, \end{array}$$

(107) $$\begin{array}{ccc} G^{2}{}_{2} & = & G^{3}{}_{3}=-\frac{m_{rr}}{r}, \end{array}$$

where the subscripts indicate differentiation with respect to the variables *v* and r. Combining the system (105)–(107) with the field equations $G^{a}{}_{b}=8\pi T^{a}{}_{b}$ yields the energy momentum tensor

(108) $$T_{ab}=T_{ab}^{\left(n\right)}+T_{ab}^{\left(m\right)},$$

where:

$$\begin{array}{ccc} T_{ab}^{\left(n\right)} & = & \zeta L_{a}L_{b},\\ T_{ab}^{\left(m\right)} & = & \left(\tilde{\rho }+P\right)\left(L_{a}N_{b}+L_{b}N_{a}\right)+Pg_{ab}. \end{array}$$

In the above,

$$L_{a}=\delta^{0}{}_{a},\;N_{a}=\frac{1}{2}\left[1-\frac{2m(v,r)}{r}\right]\delta^{0}{}_{a}-\varepsilon \delta^{1}{}_{a},$$

with $L_{c}L^{c}=N_{c}{}^{c}=0$ and $L_{c}N^{c}=-1$. The null vector $L^{a}$ is a double null eigenvector of the energy momentum tensor (108). Therefore we can write

(109) $$\begin{array}{ccc} 8\pi \zeta & = & 2\frac{m_{v}}{\varepsilon r^{2}}, \end{array}$$

(110) $$\begin{array}{ccc} 8\pi \tilde{\rho } & = & \frac{2m_{r}}{r^{2}}, \end{array}$$

(111) $$\begin{array}{ccc} 8\pi P & = & -\frac{m_{rr}}{r}, \end{array}$$

which describe the gravitational behaviour of null radiation and an additional string fluid [60,61]. The expression $T_{ab}^{\left(n\right)}$ is the component of the matter distribution, which moves along null hypersurfaces. When $\tilde{\rho }=P=0$, the equations reduce to the Vaidya solution for m=m(v). Therefore, the energy momentum tensor (108) is a generalisation of the Vaidya solution.

We define the four vectors

$$\begin{array}{ccc} E_{\left(0\right)}^{a} & = & \frac{L^{a}+N^{a}}{\sqrt{2}},\;E_{\left(1\right)}^{a}=\frac{L^{a}-N^{a}}{\sqrt{2}},\\ E_{\left(2\right)}^{a} & = & \frac{1}{r}\delta^{a}{}_{2},\;E_{\left(3\right)}^{a}=\frac{1}{r\sin\theta }\delta^{a}{}_{3}, \end{array}$$

which form a tetrad basis. A brief discussion on the derivation of the tetrad basis for Type I and Type II fluids is given in the Appendix A and Appendix B. With the above tetrad we can project the energy momentum tensor (108) to the orthonormal basis and this gives

(112) $$\begin{array}{c} T_{\left(ij\right)}=\left(\begin{array}{cccc} \frac{1}{2}\zeta +\tilde{\rho } & \frac{1}{2}\zeta & 0 & 0\\ \frac{1}{2}\zeta & \frac{1}{2}\zeta -\tilde{\rho } & 0 & 0\\ 0 & 0 & P & 0\\ 0 & 0 & 0 & P \end{array}\right). \end{array}$$

The energy momentum tensor of a Type II fluid admits one eigenvector that is doubly null, i.e., two eigenvalues will take on the same value. This matrix of eigenvalues is *not* diagonalisable [13]. The full energy conditions for the Type II fluid are then given by the following:

1. The null energy condition:
  (113) $$\zeta >0,\;\tilde{\rho }+P\geq 0.$$
2. The weak energy condition:
  (114) $$\zeta >0,\;\tilde{\rho }\geq 0,\;\tilde{\rho }+P\geq 0.$$
3. The strong energy condition:
  (115) $$\zeta >0,\;\tilde{\rho }+P\geq 0,\;P\geq 0.$$
4. The dominant energy conditions:
  (116) $$\zeta >0,\;\tilde{\rho }\geq \left|P\right|\left(\geq 0\right).$$

These conditions will hold for ζ≠0 and any proper choice of the mass function m(v,r).

In the special case when m=m(v) the solutions all reduce to the Vaidya solution and all of the energy conditions reduce to the single restriction

(117) ζ>0.

If m=m(r), then ζ=0 and the energy momentum tensor becomes a diagonal (or diagonalisable) matrix of the eigenvalues, and hence we have a Type I fluid. The energy conditions in this case are then

1. The null energy condition:
  (118) $$\tilde{\rho }+P\geq 0.$$
2. The weak energy conditions:
  (119) $$\tilde{\rho }\geq 0,\;\tilde{\rho }+P\geq 0.$$
3. The strong energy conditions:
  (120) $$P\geq 0,\;\tilde{\rho }+P\geq 0.$$
4. The dominant energy conditions:
  (121) $$\tilde{\rho }\geq 0,\;-\tilde{\rho }\leq P\leq \tilde{\rho }.$$

These conditions are analogous with (28)–(31).

## 6. Discussion

In this paper we have studied the energy conditions arising from a generalised composite relativistic fluid. Firstly, the spacetime was assumed to be spherically symmetric and the matter distribution was taken to be a viscous and anisotropic Type I field. The energy conditions were generated as a system of six equations. These contain all of the previous treatments of shearing and shear-free fluids conducting heat in the form of a radial heat flow. Secondly, a composite matter field (which was shown to be of Type I) was considered, containing a barotropic fluid, null dust and the null string. The energy conditions were found for this fluid; these can be written as a system of six equations. As far as we are aware, an analysis of the energy conditions for a composite matter distribution has not been undertaken before. Finally, a summary of the energy conditions for the two-component Type II fluid was presented, where it was shown that the reduction to those of the Type I fluid is possible only under certain conditions. An important application of the energy conditions is a radiating star in general relativity: the interior matter distribution is a Type I fluid and the exterior matter is a Type II fluid.

Further insights into the spacetime geometry will be obtained when we apply the energy conditions to particular radiating stellar models together with the complexity factor [1,2,3,4,5,6,7,8,9,10,11].

## Data Availability

This manuscript has no associated data.

## Appendix A. Orthonormal Basis: Type I Fluid

The orthornormal basis is one in which all of the vectors are unit and orthogonal to each other. We define the tetrad (or verbien) $\left\{E^{0},E^{1},E^{2},E^{3}\right\}$ with components $\left(E_{a}^{\left(0\right)},E_{a}^{\left(1\right)},E_{a}^{\left(2\right)},E_{a}^{\left(3\right)}\right)$ such that

(A1) $$\eta_{ij}=g_{ab}E_{\left(i\right)}^{a}E_{\left(j\right)}^{b},$$

relating the vector components of the tetrad with the metric $g_{ab}$. Projecting the energy momentum tensor $T_{ab}$ into the orthonormal basis can be achieved by using

(A2) $$T_{\left(ij\right)}=E_{\left(i\right)}^{a}E_{\left(j\right)}^{b}T_{ab}.$$

In the above $\eta_{ij}$ is the metric for Minkowski space. Utilising the expression (A1) along with the metric (1) yields the following vectors:

(A3) $$\begin{array}{c} E_{\left(0\right)}^{a}=\frac{1}{A}\delta^{a}{}_{0},\;E_{\left(1\right)}^{a}=\frac{1}{B}\delta^{a}{}_{1},\\ E_{\left(2\right)}^{a}=\frac{1}{C}\delta^{a}{}_{2},\;E_{\left(3\right)}^{a}=\frac{1}{C\sin\theta }\delta^{a}{}_{3}. \end{array}$$

Projecting onto the energy momentum tensor (62), using (A2) yields

$$\begin{array}{c} T_{\left(ij\right)}=\left(\begin{array}{cccc} \mu +\epsilon +\rho & -\left(qB+\epsilon \right) & 0 & 0\\ -\left(qB+\epsilon \right) & p_{\left|\right|}+\epsilon -\rho -2\eta \sigma^{1}{}_{1} & 0 & 0\\ 0 & 0 & p_{⊥}+P-2\eta \sigma^{2}{}_{2} & 0\\ 0 & 0 & 0 & p_{⊥}+P-2\eta \sigma^{2}{}_{2} \end{array}\right), \end{array}$$

in the orthonormal basis. The above matrix has off-diagonal components; however, it can be diagonalised by performing a local Lorentz boost in the (01)-plane where *v* is the velocity. If we consider the parametrisation

$$\cosh\alpha =\frac{1}{\sqrt{1-v^{2}}},\;\sinh\alpha =\frac{v}{\sqrt{1-v^{2}}},$$

the boost then gives

$$\begin{array}{ccc} {\bar{E}}_{\left(0\right)}^{a}\frac{\partial }{\partial x^{a}} & = & \left(\cosh\alpha E_{\left(0\right)}^{a}-\sinh\alpha E_{\left(1\right)}^{a}\right)\frac{\partial }{\partial x^{a}} \end{array}$$

(A4) $$\begin{array}{ccc} & = & \cosh\alpha \frac{1}{A}\frac{\partial }{\partial t}-\sinh\alpha \frac{1}{B}\frac{\partial }{\partial r},\\ {\bar{E}}_{\left(1\right)}^{a}\frac{\partial }{\partial x^{a}} & = & \left(\cosh\alpha E_{\left(1\right)}^{a}-\sinh\alpha E_{\left(0\right)}^{a}\right)\frac{\partial }{\partial x^{a}} \end{array}$$

(A5) $$\begin{array}{ccc} & = & \cosh\alpha \frac{1}{B}\frac{\partial }{\partial t}-\sinh\alpha \frac{1}{A}\frac{\partial }{\partial r}. \end{array}$$

With the above basis vectors (A4) and (A5), we now have

(A6) $$\begin{array}{ccc} T_{\left(00\right)} & = & -\frac{1}{v^{2}-1}\left(\frac{1}{A^{2}}T_{00}-2v\frac{1}{A}\frac{1}{B}T_{01}+v^{2}\frac{1}{B^{2}}T_{11}\right), \end{array}$$

(A7) $$\begin{array}{ccc} T_{\left(01\right)} & = & \frac{1}{v^{2}-1}\left(v\frac{1}{A^{2}}T_{00}-\left(1+v^{2}\right)\frac{1}{A}\frac{1}{B}T_{01}+v\frac{1}{B^{2}}T_{11}\right), \end{array}$$

(A8) $$\begin{array}{ccc} T_{\left(11\right)} & = & -\frac{1}{v^{2}-1}\left(v^{2}\frac{1}{A^{2}}T_{00}-2v\frac{1}{A}\frac{1}{B}T_{01}+\frac{1}{B^{2}}T_{11}\right). \end{array}$$

If we have $T_{\left(01\right)}=0$, then there exists the following restriction for *v* from (A7):

(A9) $$v=\frac{1}{2T_{01}}AB\left(\frac{1}{A^{2}}T_{00}+\frac{1}{B^{2}}T_{11}\pm \Delta \right).$$

The energy momentum tensor $T_{\left(ij\right)}$ is Type I only in the range −1<v<1. In the region v=±1, $T_{\left(ij\right)}$ is a Type II distribution. In the region |v|>1, two eigenvalues will become complex and $T_{\left(ij\right)}$ will be a Type IV fluid. In the above equations we note that

$$\begin{array}{ccc} \Delta^{2} & = & {\left(\frac{1}{A^{2}}T_{00}+\frac{1}{B^{2}}T_{11}\right)}^{2}-4\left(\frac{1}{A^{2}}\frac{1}{B^{2}}\right)T_{01}^{2}\\ & = & {\left(\mu +p_{\left|\right|}+2\epsilon -2\eta \sigma^{1}{}_{1}\right)}^{2}-4{\tilde{q}}^{2}, \end{array}$$

where $\tilde{q}=qB+\epsilon$. Substituting Equation (A9) into the expressions (A6) and (A8) yields

$$\begin{array}{ccc} T_{\left(00\right)} & = & \frac{1}{2}\left(\frac{1}{A^{2}}T_{00}-\frac{1}{B^{2}}T_{11}\mp \Delta \right)\\ & = & \frac{1}{2}\left(\mu -p_{\left|\right|}+2\rho +2\eta \sigma^{1}{}_{1}\mp \Delta \right),\\ T_{\left(11\right)} & = & -\frac{1}{2}\left(\frac{1}{A^{2}}T_{00}-\frac{1}{B^{2}}T_{11}\pm \Delta \right)\\ & = & -\frac{1}{2}\left(\mu -p_{\left|\right|}+2\rho +2\eta \sigma^{1}{}_{1}\pm \Delta \right). \end{array}$$

Therefore, we have diagonalised $T_{\left(ij\right)}$ with

$$T_{\left(ij\right)}=diag\left(T_{\left(00\right)},T_{\left(11\right)},T_{\left(22\right)},T_{\left(33\right)}\right),$$

and so the energy conditions (92)–(98) may be utilised for the Type I fluid.

## Appendix B. Orthonormal Basis: Type II Fluid

When considering a Type II fluid, transforming to an orthonormal basis is less trivial than for a Type I distribution. Projection of the energy momentum tensor (108) into the orthonormal basis can be achieved using (A2), which gives

$$\begin{array}{c} T_{\left(ij\right)}=\left(\begin{array}{cccc} \frac{1}{2}\zeta +\tilde{\rho } & \frac{1}{2}\zeta & 0 & 0\\ \frac{1}{2}\zeta & \frac{1}{2}\zeta -\tilde{\rho } & 0 & 0\\ 0 & 0 & P & 0\\ 0 & 0 & 0 & P \end{array}\right), \end{array}$$

from (112). This calculation yields the vectors

(A10) $$\begin{array}{ccc} E_{\left(0\right)}^{a} & = & \left(\frac{-1}{\sqrt{2}},\frac{-1}{\sqrt{2}}\left(1-\frac{1}{2}\left(1-\frac{2m}{r}\right)\right),0,0\right), \end{array}$$

(A11) $$\begin{array}{ccc} E_{\left(1\right)}^{a} & = & \left(\frac{1}{\sqrt{2}},\frac{-1}{\sqrt{2}}\left(1+\frac{1}{2}\left(1-\frac{2m}{r}\right)\right),0,0\right), \end{array}$$

(A12) $$\begin{array}{ccc} E_{\left(2\right)}^{a} & = & \left(0,0,\frac{1}{r},0\right), \end{array}$$

(A13) $$\begin{array}{ccc} E_{\left(3\right)}^{a} & = & \left(0,0,0,\frac{1}{r\sin\theta }\right). \end{array}$$

We note that the above can then be written, using (108) and the null vectors $L^{a}$ and $N^{a}$, as

$$\begin{array}{ccc} E_{\left(0\right)}^{a} & = & \frac{L^{a}+N^{a}}{\sqrt{2}},\\ E_{\left(1\right)}^{a} & = & \frac{L^{a}-N^{a}}{\sqrt{2}},\\ E_{\left(2\right)}^{a} & = & \frac{1}{r}\delta^{a}{}_{2},\\ E_{\left(3\right)}^{a} & = & \frac{1}{r\sin\theta }\delta^{a}{}_{3}, \end{array}$$

in the orthonormal basis. It can be seen that the energy momentum tensor for a Type II fluid in the orthonormal basis is *not* diagonalisable.
